# Loss to follow-up in a population-wide brief contact intervention to prevent suicide attempts - The VigilanS program, France

**DOI:** 10.1371/journal.pone.0263379

**Published:** 2022-03-01

**Authors:** Larissa Djembi Fossi, Christophe Debien, Anne-Laure Demarty, Guillaume Vaiva, Antoine Messiah

**Affiliations:** 1 INSERM, MOODS Research unit “Depression, Anxiety, Psychotraumatism and Suicide”, Centre de recherche en Epidémiologie et santé des populations (CESP), Université Paris-Saclay, Hôpital Paul-Brousse, Villejuif, France; 2 INSERM, Sorbonne University, Institut Pierre Louis d’Épidémiologie et de Santé Publique, Social Epidemiology Team, Paris, France; 3 Department of Psychiatry, University Hospital of Lille, Lille, France; 4 Univ. Lille, Inserm, CHU Lille, U1172—LilNCog (JPARC)—Lille Neurosciences & Cognition, Lille, France; 5 Centre National de Ressources et Résilience Pour Les Psychotraumas (Cn2r Lille Paris), Department of Psychiatry, University Hospital of Lille, Lille, France; Semmelweis University: Semmelweis Egyetem, HUNGARY

## Abstract

**Background:**

Brief Contact Interventions (BCIs) after a suicide attempt (SA) are an important element of prevention against SA and suicide. VigilanS generalizes to a whole French region a BCI combining resource cards, telephone calls and sending postcards, according to a predefined algorithm. However, a major obstacle to such real-life intervention is the loss of contact during follow-up. Here, we analyze the occurrence of loss of follow-up (LFU) and compare characteristics of patients LFU with follow-up completers.

**Methods:**

The study concerned patients included in VigilanS over the period from 1^st^ January 2015 to 31 December 2018, with an end of follow-up on 1^st^ July 2019. We performed a series of descriptive analysis and logistic regressions. The outcome was the loss to follow-up, relative to the 6th month call marking the end of the follow-up; the predictive variables were the characteristics of the patient at entry and during follow-up. Age and sex were considered as adjustment variables.

**Results:**

11879 inclusions occurred during the study period, corresponding to 10666 different patients. The mean age was 40.6 ± 15 years. More than a third were non-first suicide attempters (46.6%) and the most frequent means of suicide was by voluntary drug intoxication (83.2%). 8335 patients were LFU. After simple and multiple regression, a significant relationship with loss to follow-up was identified among non-first suicide attempters, alcohol consumers, patients having no companion on arrival at the emergency room, patients who didn’t make or receive any calls. An increased stay in hospital after a SA was a protective factor against loss of follow-up.

**Conclusion:**

A majority of patients were lost to follow-up by the expected surveillance time of 6 months. Characteristics of lost patients will help focusing efforts to improve retention in the VigilanS program and might give insights for BCI implemented elsewhere.

## Introduction

Suicide attempt (SA) is one of the major public health problems, as well as one of the most important indicators of mental health [1]. According to WHO, SA refers to all non-lethal suicidal behavior and to an act of self-intoxication, self-harm or self-injury with intent to die or not [2]. SA are nearly 20 times more common than suicide deaths [3], and history of SA is predictive of subsequent attempts and risk of death by suicide (which typically occurs after several repeated attempts) [3, 4]. The risk of completed suicide for people who have already attempted suicide is 40 to over 100 times higher than that of the general population [5, 6], and the risk of recurrence is highest immediately after discharge from hospital, with one in three patients repeating the attempt within 30 days [7]. Faced with the very high risk of suicide after a SA, many researchers have tried Brief Contact Interventions (BCI) on patients admitted to hospital after a SA [8, 9].

These BCIs include: "phone calls" focusing on patient’s mental health state and adherence to post-discharge treatment [10], “issuing a resource card” giving a phone number of a crisis management professional [11], "Sending letters" from a person who has met the suicidal patient during his/her hospital stay [12], "Sending postcards" [13], and "sms" consisting in maintaining contact through text messages [14]. Several researchers have shown the effectiveness of these BCIs in reducing suicidal behaviour. This is the case of Fleishmann et al who found a significant reduction in the number of SA in their study, based on continuous communication in combination with usual treatments [15], Bertolote et al in 2010 found an effectiveness of telephone calls on suicide mortality in their study [16], as well as Cebria et al in 2013 in Spain, showing a decrease in the number of SA reccurence related to phone calls [17]. A randomized controlled trial was realized on more than 1000 patients in 24 hospitals in France, comparing Algos, an algorithm that combined different types of BCIs into a single operational monitoring system, to treatment as usual. Results from this trial led the authors and health care authorities to scale it up to the general population. Given some equivocal results from the Algos trial [18, 19], the intervention was significantly enhanced, and relabeled VigilanS (Vigilance for the prevention of Suicide recurrence).

Created in 2014 in collaboration with the Nord-Pas de Calais hospitals, and operational since 2015, VigilanS allows to recontact any suicidal person immediately after a SA, by a team of mental health care professionals specially trained in suicidal crisis management [20]. It is a region wide BCI. According to the study by Fossi Djembi et al and Vaiva et al, VigilanS can be an effective system for SA reduction [21, 22].

A major barrier to follow-up studies, however, is the loss of contact during follow-up. In some clinical studies, it has been found that 50% or more of patients did not show up for treatment or withdrew their participation within a week [23, 24].

In studies of brief contact interventions in real world conditions, such as Lewis et al’s study of a brief intervention for weight management in primary care, patients were considered as lost to follow-up after a maximum of three unsuccessful contact attempts, using multiple means and trying at different times. According to Levi’s et al, more than this could be seen as harassment [25]. Vaiva et al also highlighted follow-up issues in their Algos study, and noted that results may be distorted by excessive lost to follow-up [18]. According to the study by Gysin-Maillart et al, the majority of follow-up losses occurred during the 1–12 month period, as opposed to the 12–25 month period, regardless of the subject group [26].

The management of patients lost to follow-up in analyses often involves sensitivity analyses, to estimate what would happen to these lost patients if they were still present until the end of the study. This is the case in the studies of Stead et al, and Lai et al, who simply considered the lost to follow-up as regular smokers in their studies of smoking cessation interventions [27, 28].

However, these studies that included the lost to follow-up in the analysis had a low risk of bias because people lost to follow-up were similar to the other participants and in small number [29, 30].

Despite our extensive literature review, including studies with detailed description of suicidal patients, we failed to find a comparison of patients lost to follow-up against those remaining till the end of the study. The analysis of these patients is particularly useful in order to understand how results based on study completers might be distorted.

### Study objectives

The objective of our study was to analyze the course of follow-up in VigilanS, which is a full scale, region-wide BCI, implemented in real-life conditions–as opposed to the experimental conditions of a clinical trial. More specifically, our aims were to study the occurrence of loss of follow-up and compare characteristics of patients lost to follow-up with follow-up completers.

## Method

### Ethics approval and consent to participate

The authors assert that all procedures contributing to this work comply with the ethical standards of the relevant national and institutional committees on human experimentation and with the Helsinki Declaration of 1975, as revised in 2008. All procedures involving human subjects/patients were approved by the French Ministry of Health, and approved by the Comité′de Protection des Personnes of Nord-Pas-de-Calais region (Ethics Committee).

#### a- Patient selection

Our study was carried out on all patients included in VigilanS over the period from 1^st^ January 2015 to 31 December 2018 in the Nord-Pas-de-Calais region. 1^st^ July 2019 was the end of follow-up for our study. Patients who died during the follow were excluded from the analysis, as well as patients who were minors (under 18 years old).

#### b- Description of the VigilanS system

In VigilanS, every suicidal person leaving the hospital is given an information letter by a member of the team who took care of him or her, in which the term and the conditions of the system are described, including a right to object. A resource card is also handed out, giving a free regional telephone number to reach a monitoring unit which can be called in case of need, as well as an afterhours emergency number.

The regional monitoring unit is made up of psychologists or psychiatric nurses, specially trained. In VigilanS, an important component is outgoing and incoming calls. Systematic outgoing calls are issued by the VigilanS team, between the 10^th^ and 21^st^ days for non-first suicide attempters (D10-D21 call), and at the 6^th^ month (6M interview) for all patients. D10-D21 call concerns non-first suicide attempters (having already made at least one suicidal act before entering VigilanS), because they are most at risk of having a new SA during this period.

6M call concerns all suicidal subjects (first and non-first suicide attempters). They are contacted at the end of the 6^th^ month following discharge from the hospital, for a check-up. For first suicide attempters and for non-first suicide attempters who have not responded to the call D10-D21, it is sometimes the only direct contact with the monitoring unit. It is not uncommon to have to make 2 to 3 calls before being able to reach the patient. During this call, questions are asked to the patient, in order to assess whether or not further follow-up in VigilanS is necessary, depending on the patient’s condition.

Apart from these systematic two calls, intermediate calls are also placed. Intermediate calls are calls made on the initiative of vigilantes outside the 2 calls provided for by the program (the call at D10- D21 and the call at 6 months). Some monitoring unit plan intermediate calls when they are worried about the patient. A medical appointment scheduled for the patient can also be a reason for programming an intermediate call. It allows checking that the patient follows the post-discharge care program adequately.

Incoming calls are calls made by the patient to VigilanS. Some patients may call several times during their 6-months enrolment.

When patients are unreachable or when they are non-adherent to the post-discharge care, the team send them postcards. Calls from health professionals who follow patients, even if their volume increased significantly over time, remain scarce. Detailed description of the VigilanS intervention is published elsewhere [20].

A brief report is sent to the patient’s general practitioner and referring psychiatrist at admission and at each phone or in-person contact.

#### c- Data processing

Any given patient could be enrolled several times into VigilanS, in case of repeated SA with more than 6 months between them. Therefore, statistical units of analysis (SUA) could be either the SAs that triggered an inclusion in VigilanS, with possibly several records per patients, or the patients, with a unique record made of all successive inclusions when appropriate. For the current paper, SUA were patients; for those with multiple entries into VigilanS, the first one was selected, in order to compare patients at similar stages of the VigilanS intervention.

When a 6M call is missed, VigilanS further tries to get in touch with the patient through other call attempts and postcard mailings; patient’s physicians and entourage are also contacted. When a contact with the patient is successful, a 6M interview is performed. A patient is classified as lost to follow-up (LFU) if he could not be reached by a 6^th^ months call and VigilanS has no further news since the missed call.

#### d- Variables study

This study outcome was the loss to follow-up, and the explanatory variables were the characteristics of the patient at first entry and during first follow-up in VigilanS. The list of variables can be found in the appendices (**S1 Table**).

#### e- Statistical analysis

The following statistical analyses were conducted:

- **Descriptive analysis** **of the data**

Descriptive statistics of all patients’ characteristics were made, as well as descriptive statistics of first suicidal attempters and non-first suicidal attempters, and descriptive statistics of non-first suicidal attempters and whose D10-D21 call was successful. Quantitative variables were summarized by their mean and standard deviation; for qualitative variables, percentage was provided.

- **Survival analysis of 6**^**th**^ **month follow-up**

Since the 6th month follow-up interview could occur at any time after 6 months, due to VigilanS repetitive attempts to complete this interview, a survival analysis was performed, on a VigilanS record basis. This allowed estimating (1) the average delay necessary to achieve a 6-M interview, including recoveries from initial losses to follow-up; and (2) the percent of interviews achieved at any given time. The event of interest was the realization of a 6^th^ month interview, which was right-censored, and the time-to-event was the time elapsed since the index SA.

- **Simple logistic** **regression**

A Simple logistic regression was performed on all patients, then on first suicidal attempters and the non-first suicidal attempters. The aim of the simple logistic regression was to study the relationship between loss of follow-up (dependent variable) and patient characteristics (independents variables). Each variable was adjusted for age and gender, which were considered as potential confounding factors. The Chi-square test was provided for the qualitative variables, and the Student test for the qualitative variables.

- **Multiple** **logistic regression**

In order to account for possible interactions between the variables, multiple logistic regression was also performed. The variables included in the multiple logistic regression were those with a P value <0.25 resulting from the simple logistic regression. Age and gender were systematically included as adjustment variables. Odds ratios (OR) confirmed the significant relationship between the dependent and independent variables, as well as their confidence intervals (95% CI) and the P-value <5%.

The software used was software R version 3. 6. 3.

## Results

**Fig 1:**
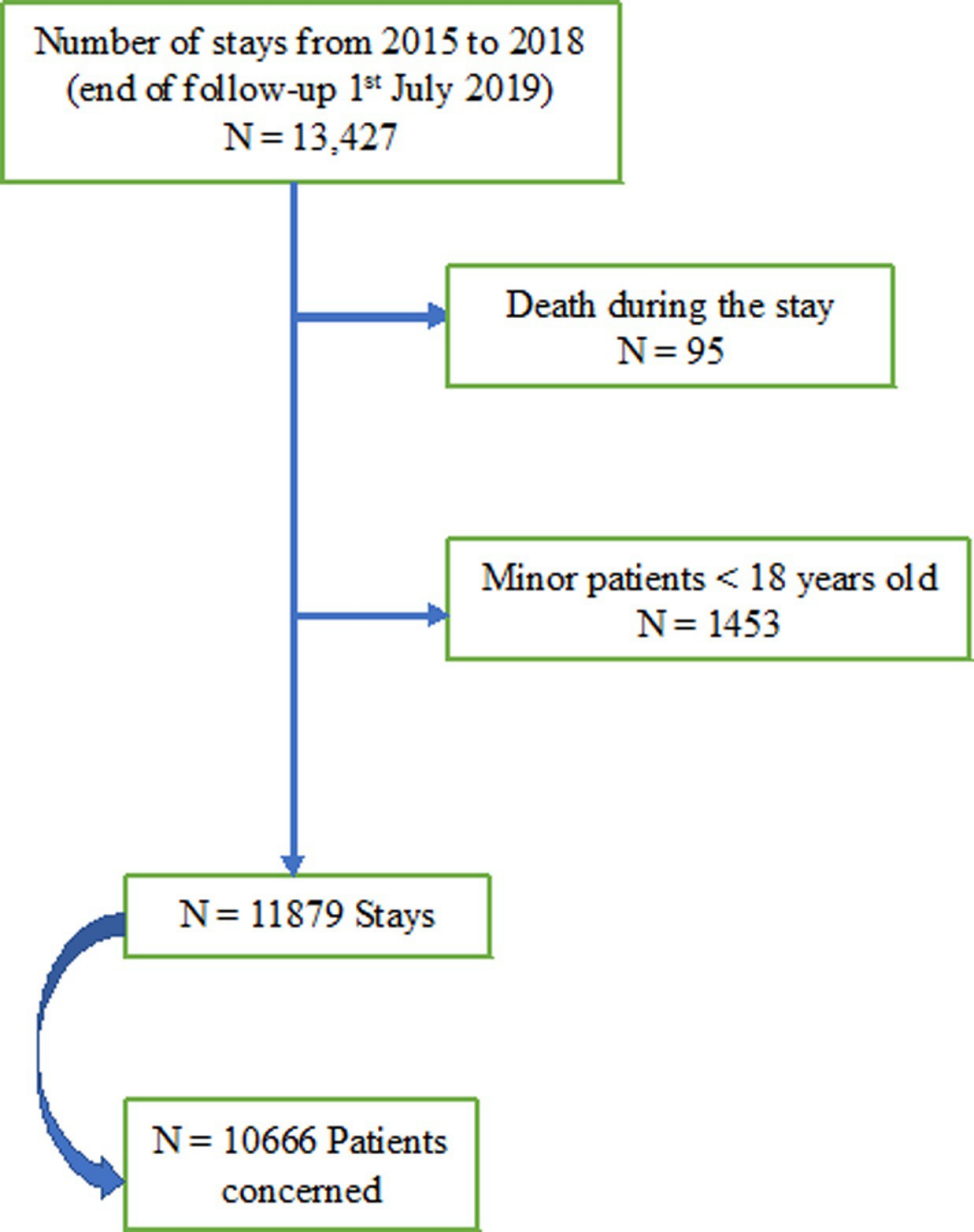
Flow chart for patient selection in the analysis.

From 1^st^ January 2015 to 31^st^ December 2018, we had 13427 stays, marked by an end of follow-up on 1^st^ July 2019. After removing the deaths during the stays and patients that were under 18 years of age, we finally had 11879 stays, corresponding to 10666 different patients **(Fig 1)**.

### I- Description of patient

#### a) General description

The minimum age at entry into VigilanS was 18 years, the maximum was 94 years, with a mean ± SD of 40.6 ± 15 years. Most patients were women (58.7%). The departments of France studied in our study are the departments of North and Pas-de-Calais, which form the sub-region of Nord-Pas-de-Calais. Most patients were from the North Department of France (54.6%). More than ¾ of patients had an accompanying person (74.8%), and the most frequent length of hospital stay was one day (48.0%). Among non-first suicide attempters, more than half of the calls to D10-D21 were successful (52.8%). However, there were few successful calls in the 6^th^ month (18.4%) overall. Few patients have had successful telephone contacts (34.6%), but some have had cards sent when the call was unsuccessful (39.7%). The most frequent means of suicide was by Voluntary Drug Intoxication (VDI; 83.2%), followed by phlebotomy (7.4%). (**Table 1**). Phlebotomy here refers to a form of venous self-mutilation by venipuncture or intravenous cannula [31, 32].

**Table 1:** Description of patients at first entry into VigilanS.

Variables	All patients (N = 10666)	First suicide attempters (N = 5700)	Non-first suicide attempters (N = 4966)
**Age**	40.6±14.7 ^a^	39.2±15.1 ^a^	42.2±14.0 ^a^
**Sex**			
Male	4404 (41.3%)	2489 (43.7%)	1915 (38.6%)
Female	6262 (58.7%)	3211 (56.3%)	3051 (61.4%)
**Geographic sub region (French “Departement”)**			
North	5828 (54.6%)	3036 (53.3%)	2792 (56.2%)
Pas de Calais	4127 (38.7%)	2271 (39.8%)	1856 (37.4%)
Other	711 (6.7%)	393 (6.9%)	318 (6.4%)
**Alcohol consumption**			
No	5187 (48.6%)	2938 (51.5%)	2249 (45.3%)
Yes	5479 (51.4%)	2762 (48.5%)	2717 (54.7%)
**Accompanying person**			
No	2688 (25.2%)	1195 (21.0%)	1493 (30.1%)
Yes	7978 (74.8%)	4505 (79.0%)	3473 (69.9%)
**Duration of hospitalization stay (days)**			
0	1522 (14.3%)	842 (14.8%)	680 (13.7%)
1	5120 (48.0%)	2801 (49.1%)	2319 (46.7%)
2+	4024 (37.7%)	2057 (36.1%)	1967 (39.6%)
**Outgoing D10-D21 call issued successfully?**			
No	-	Not concerned	2342 (47.2%)
Yes	-	Not concerned	2624 (52.8%)
**Number of intermediate outgoing calls issued successfully**			
0	9797 (91.9%)	5534 (97.1%)	4263 (85.8%)
1+	869 (8.1%)	166 (2.9%)	703 (14.2%)
**Number of incoming calls from the patient**			
0	9134 (85.6%)	5346 (93.8%)	3788 (76.3%)
1+	1532 (14.4%)	354 (6.2%)	1178 (23.7%)
**Phone contact**			
In contact (*outgoing calls issued successfully or incoming calls*)	3687 (34.6%)	409 (7.2%)	3278 (66.0%)
No contact but cards send	4235 (39.7%)	3201 (56.2%)	1034 (20.8%)
No contacts No cards send	2744 (25.7%)	2090 (36.7%)	654 (13.2%)
**Outgoing 6M call issued successfully?**			
No	8699 (81.6%)	4680 (82.1%)	4019 (80.9%)
Yes	1967 (18.4%)	1020 (17.9%)	947 (19.1%)
**Number of outgoing call to the patient’s family and friends**			
0	9437 (88.5%)	5386 (94.5%)	4051 (81.6%)
1+	1229 (11.5%)	314 (5.5%)	915 (18.4%)
**Number of incoming call from the patient’s family and friends**			
0	10290 (96.5%)	5593 (98.1%)	4697 (94.6%)
1+	376 (3.5%)	107 (1.9%)	269 (5.4%)
**Year**			
2015	1807 (16.9%)	909 (15.9%)	898 (18.1%)
2016	2699 (25.3%)	1438 (25.2%)	1261 (25.4%)
2017	3043 (28.5%)	1655 (29.0%)	1388 (28.0%)
2018	3117 (29.2%)	1698 (29.8%)	1419 (28.6%)
***MEANS OF SA***			
**VDI**			
No	1791 (16.8%)	970 (17.0%)	821 (16.5%)
Yes	8875 (83.2%)	4730 (83.0%)	4145 (83.5%)
**Hanging**			
No	10122 (94.9%)	5349 (93.8%)	4773 (96.1%)
Yes	544 (5.1%)	351 (6.2%)	193 (3.9%)
**Phlebotomy (Self-bloodletting)**			
No	9877 (92.6%)	5313 (93.2%)	4564 (91.9%)
Yes	789 (7.4%)	387 (6.8%)	402 (8.1%)
**Others (Firearms, Lesions, Drowning, Jump)**			
No	10327 (96.8%)	5515 (96.8%)	4812 (96.9%)
Yes	339 (3.2%)	185 (3.2%)	154 (3.1%)
***VARIABLES OF D10-D21 CALLS ISSUES SUCCESSFULLY***			***(N = 2624)***
**Evolution of discomfort since SA**			
Stable	-	-	805 (30.7%)
Favorable	-	-	1721 (65.6%)
Unfavorable	-	-	98 (3.7%)
**Need help**			
No	-	-	595 (22.7%)
Yes	-	-	2029 (77.3%)
**Followed by a Psychiatrist**			
No	-	-	896 (34.1%)
Yes	-	-	1728 (65.9%)
**Patient’s state at the end of the interview**			
Good	-	-	1040 (39.6%)
Poor, not in crisis	-	-	1488 (56.7%)
In crisis	-	-	96 (3.7%)
**Postcards sent**			
No	-	-	977 (37.2%)
Yes	-	-	1647 (62.8%)

^a^ Means ± Standard deviation

#### b) Description of first suicide attempters and non-first suicide attempters

There were some slight variations between the first suicide attempters and non-first suicide attempters: the non-first suicide attempters were slightly older (42 years) and more female (61.4%) than the first suicide attempters (39 years; women 56.3%). As regards alcohol consumption, this refers to those who did or did not consume alcohol during their suicide attempt. More of non-first suicide attempters consume alcohol than first suicide attempters. Most of the first suicide attempters were accompanied on arrival at the emergency room (79.0%), but fewer first suicide attempters had successful telephone contacts during the follow-up (7.2%), unlike the non-first suicide attempters (66.0%).

#### c) Description of D10-D21 calls

This description concerns non-first suicide attempters who were reached successfully by the D10-D21 call. The evolution of discomfort since SA was in majority favorable (65.6%). More than ¾ needed help (77.3%), and most had a follow-up by a psychiatrist during their VigilanS enrollment (65.9%). At the end of the interview, most patients were still in distress (56.7%). The sending of cards was programmed for most patients after the D10-D21 call (62.8%), in order to maintain contact (**Table 1**).

#### d) 6th month call

**Fig 2:**
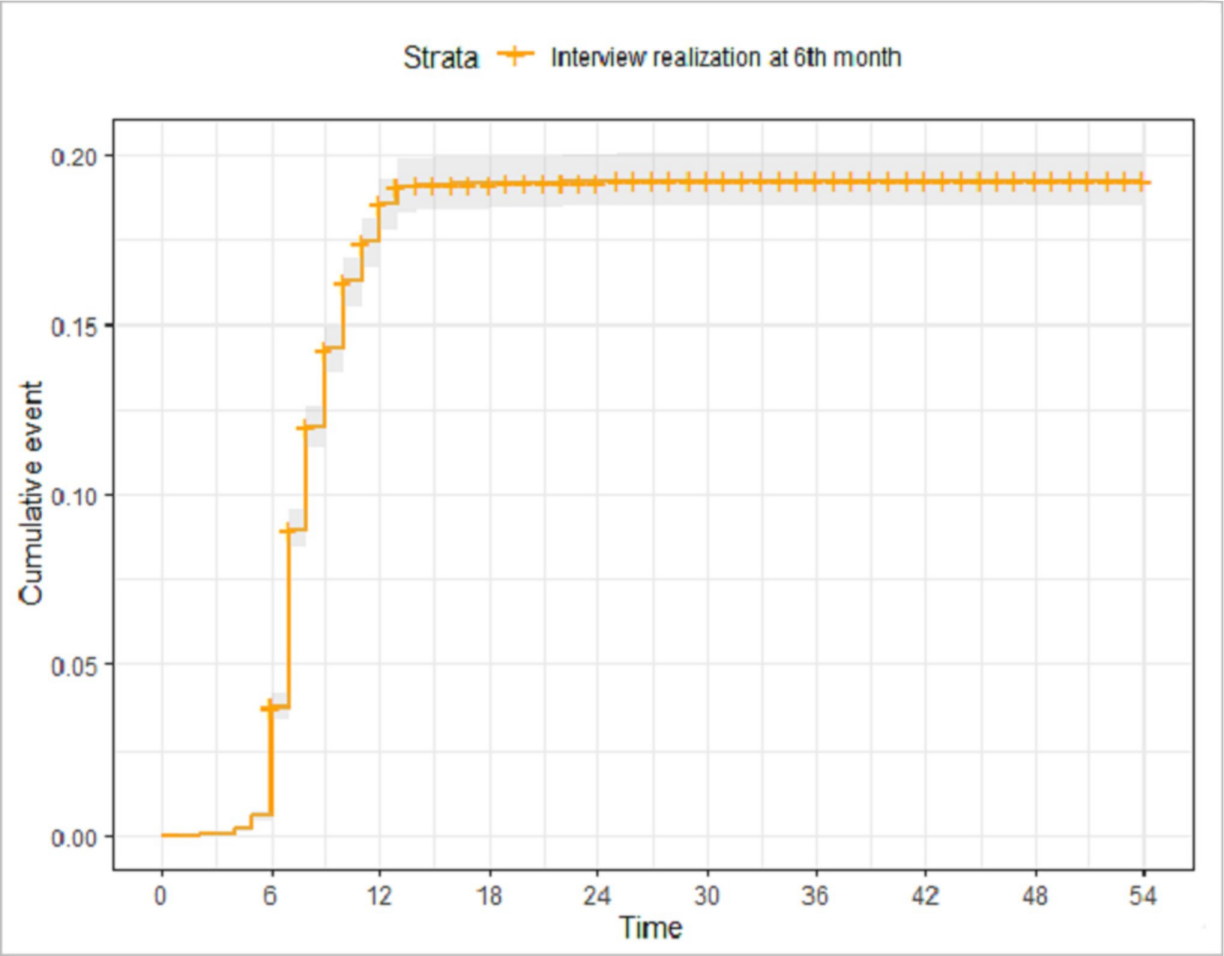
Interview realization survival analysis at 6M as a function of follow-up time in months.

**Fig 2** shows the survival analysis for the “6-month interview” event, according to the duration of the follow-up in months. The aim here was to assess the time it took to realize the interview at the end of the follow-up in VigilanS. It was found that most of the interviews carried out were not done at the end of the 6 months as planned, but later. The mean time for this interview was around 8 months.

### II- Analysis of lost to follow-up (LF) and non-lost to follow-up (NLF)

#### a) Simple logistic regression

In total, 78% of patients were LFU and 22% of patients were Non-LFU in the first stay (**S1 Fig**).

After adjusting for age and sex, it was found a significant relationship between lost to follow-up and suicide attempters (non-first suicide attempters), alcohol consumers, duration of hospitalization stay (at least one day in hospitals), number of outgoing calls, incoming calls and phone contacts (no calls), the year of entry into VigilanS. There was also a significant relationship between the method used for SA (VDI) and the risk of LFU. **Table 2.**

**Table 2:** Comparison of general characteristics of lost to follow-up and non-lost to follow-up patients and simple age and sex-adjusted logistic regression.

Variables	All patients		
	LFU (N = 8335)	Non-LFU (N = 2331)	P (X2)
**Age**	39.9±14.6	42.9±14.7	
**Sex**			
Male	3503 (42.0%)	901 (38.6%)	
Female	4832 (58.0%)	1430 (61.4%)	
**Geographic sub region (French “Departement”)**			
North	4516 (54.2%)	148 (56.3%)	
Pas De Calais	3256 (39.1%)	1312 (37.4%)	0.170
Others *	563 (6.7%)	871 (6.3%)	
**Suicide attempters**			
Non-first suicide attempters	3773 (45.3%)	1193 (51.2%)	4*10 ^**−5**^
First suicide attempters *	4562 (54.7%)	1138 (48.8%)	
**Alcohol consumption**			
No *	4009 (48.1%)	1178 (50.5%)	
Yes	4326 (51.9%)	1153 (49.5%)	**0.017**
**Accompanying person**			
No	2128 (25.5%)	560 (24.0%)	
Yes *	6207 (74.5%)	1171 (76.0%)	0.10
**Duration of hospitalization stay**			
0 *	1232 (14.8%)	290 (12.4%)	
1	4049 (48.6%)	1071 (46.0%)	**0.001**
2+	3054 (36.6%)	970 (41.6%)	
**Number of outgoing call issued succesfully**			
0	7788 (93.4%)	2009 (86.2%)	<2*10 ^**−16**^
1+ *	547 (6.6%)	322 (13.8%)	
**Number of incoming calls from the patient**			
0	7365 (88.4%)	1769 (75.9%)	
1+ *	970 (11.6%)	562 (24.1%)	<2*10 ^**−16**^
**No phone contacts?**			
In contact (*outgoing calls issued successfully or incoming calls*) *	2580 (31.0%)	1107 (47.5%)	
No contact but cards send	3476 (41.7%)	759 (32.6%)	<2*10 ^**−16**^
No contacts No cards send	2279 (27.3%)	465 (19.9%)	
**Number of outgoing calls to the patient’s family and friends**			
0	7465 (89.6%)	1972 (84.6%)	
1+ *	870 (10.4%)	359 (15.4%)	9*10 ^**−11**^
**Number of incoming calls from the patient’s family and friends**			
0	8083 (97.0%)	2207 (94.7%)	
1+ *	252 (3.0%)	124 (5.3%)	9*10 ^**−7**^
**Year**			
2015 *	1170 (14.0%)	637 (27.3%)	
2016	1908 (22.9%)	791 (33.9%)	<2*10 ^**−16**^
2017	2690 (32.3%)	353 (15.2%)	
2018	2567 (30.8%)	550 (23.6%)	
***MEANS OF SA***			
**VDI**			
No *	1446 (17.4%)	345 (14.8%)	
Yes	6889 (82.6%)	1986 (85.2%)	**0.023**
**Hanging**			
No *	7895 (94.7%)	2227 (95.5%)	
Yes	440 (5.3%)	104 (4.5%)	0.224
**Phlebotomy (Self-bloodletting)**			
No *	7724 (92.7%)	2153 (92.4%)	
Yes	611 (7.3%)	178 (7.6%)	0.294
**Others (Firearms, Lesions, Drowning, Jump)**			
No *	8067 (96.8%)	2260 (97.0%)	
Yes	268 (3.2%)	71 (3.0%)	0.923

However, certain variables are significant among first suicide attempters but not among non-first suicide attempters, and inversely. This is the case, for example, with variables such as the non-presence of a companion on arrival at the emergency room and the number of calls made to friends and family (no calls), which are significant for first suicide attempters and non-significant for non-first suicide attempters. Inversely, variables such as department, alcohol and duration of hospitalization stay are significant in the case of non-first suicide attempters and non-significant in the case of first suicide attempters **Table 3.**

**Table 3:** Comparison of general characteristics of lost to follow-up and non-lost to follow-up patients and simple age and sex-adjusted logistic regression.

Variables	First suicide attempters			Non-first suicide attempters		
	LFU (N = 4562)	Non-LFU (N = 1138)	P (X2)	LFU (N = 3773)	Non-LFU (N = 1193)	P (X2)
**AGE**	38.5±14.9	42.0±15.4		**41.7±14.0**	**43.7±13.9**	
**SEX**						
Male	2025 (44.4%)	464 (40.8%)		1478 (39.2%)	437 (36.6%)	
Female	2537 (55.6%)	674 (59.2%)		2295 (60.8%)	756 (63.4%)	
**Geographic sub region (French “Departement”)**						
North	2435 (53.4%)	601 (52.8%)		2081 (55.1%)	711 (59.6%)	
Pas De Calais	1805 (39.6%)	466 (41.0%)	0.574	1451 (38.5%)	405 (34.0%)	**0.018**
Others *	322 (7.0%)	71 (6.2%)		241 (6.4%)	77 (6.4%)	
**Alcohol consumption**						
No *	2330 (51.1%)	608 (53.4%)		1679 (44.5%)	570 (47.8%)	
Yes	2232 (48.9%)	530 (46.6%)	0.087	2094 (55.5%)	623 (52.2%)	**0.041**
**Accompanying person**						
No	981 (21.5%)	214 (18.8%)		1147 (30.4%)	346 (29.0%)	
Yes *	3581 (78.5%)	924 (81.2%)	**0.027**	2626 (69.6%)	847 (71.0%)	0.384
**Duration of hospitalization stay (days)**						
0 *	678 (14.9%)	164 (14.4%)		554 (14.7%)	126 (10.5%)	
1	2282 (50.0%)	519 (45.6%)	0.051	1767 (46.8%)	552 (46.3%)	9*10 ^**−4**^
2+	1602 (35.1%)	455 (40.0%)		1452 (38.5%)	515 (43.2%)	
**Number of outgoing call issued**						
0	4471 (98.0%)	1063 (93.4%)		3317 (87.9%)	946 (79.3%)	
1+ *	91 (2.0%)	75 (6.6%)	2*10 ^**−12**^	456 (12.1%)	247 (20.7%)	4*10 ^**−12**^
**Number of incoming calls from the patient**						
0	4391 (96.3%)	955 (83.9%)		2974 (78.8%)	814 (68.2%)	
1+ *	171 (3.7%)	183 (16.1%)	<2*10 ^**−16**^	799 (21.2%)	379 (31.8%)	7*10 ^**−12**^
**No phone contacts?**						
In contact (*outgoing calls issued successfully or incoming calls*) *	2620 (57.4%)	581 (51.0%)		856 (22.7%)	178 (14.9%)	
No contact but cards send	1734 (38.0%)	356 (31.3%)	<2*10 ^**−16**^	545 (14.4%)	109 (9.1%)	2*10 ^**−14**^
No contacts No cards send	208 (4.6%)	201 (17.7%)		2372 (62.9%)	906 (76.0%)	
**Number of outgoing calls to the patient’s family and friends**						
0	4373 (95.9%)	1013 (89.0%)		3092 (81.9%)	959 (80.4%)	
1+ *	189 (4.1%)	125 (11.0%)	<2*10 ^**−16**^	681 (18.1%)	234 (19.6%)	0.174
**Number of incoming calls from the patient’s family and friends**						
0	4492 (98.5%)	1101 (96.7%)		3591 (95.2%)	1106 (92.7%)	
1+ *	70 (1.5%)	37 (3.3%)	8*10 ^**−4**^	182 (4.8%)	87 (7.3%)	**0.001**
**Year**						
2015 *	622 (13.6%)	287 (25.2%)		548 (14.5%)	350 (29.4%)	
2016	1128 (24.7%)	310 (27.2%)	<2*10 ^**−16**^	780 (20.7%)	481 (40.3%)	<2*10 ^**−16**^
2017	1480 (32.5%)	175 (15.4%)		1210 (32.1%)	178 (14.9%)	
2018	1332 (29.2%)	366 (32.2%)		1235 (32.7%)	184 (15.4%)	
***MEANS OF SA***						
**VDI**						
No *	803 (17.6%)	167 (14.7%)		643 (17.0%)	178 (14.9%)	
Yes	3759 (82.4%)	971 (85.3%)	0.058	3130 (83.0%)	1015 (85.1%)	0.175
**Hanging**						
No *	4277 (93.7%)	1072 (94.2%)		3618 (95.9%)	1155 (96.8%)	
Yes	285 (6.3%)	66 (5.8%)	0.636	155 (4.1%)	38 (3.2%)	0.268
**Phlebotomy (Self-bloodletting)**						
No *	4243 (93.0%)	1070 (94.0%)		3481 (92.3%)	1083 (90.8%)	
Yes	319 (7.0%)	68 (6.0%)	0.462	292 (7.7%)	110 (9.2%)	0.059
**Others (Firearms, Lesions, Drowning, Jump)**						
No *	4413 (96.7%)	1102 (96.8%)		3654 (96.8%)	1158 (97.1%)	
Yes	149 (3.3%)	36 (3.2%)	0.924	119 (3.2%)	35 (2.9%)	0.817
***VARIABLES OF D10-D21 CALLS ISSUES SUCCESSFULLY***				***(LFU = 1891)***	***(Non-LFU = 733)***	
**Evolution of discomfort since SA**						
Stable	-	-		570 (30.1%)	235 (32.1%)	
Favorable *	-	-		1257 (66.5%)	464 (63.3%)	0.189
Unfavorable	-	-		64 (3.4%)	34 (4.6%)	
**Need help**						
No *	-	-		409 (21.6%)	186 (25.4%)	
Yes	-	-		1482 (78.4%)	547 (74.6%)	**0.034**
**Followed by a Psychiatrist**						
No *	-	-		653 (34.5%)	243 (33.1%)	
Yes	-	-		1238 (65.5%)	490 (66.9%)	0.529
**Patient’s state at the end of the interview**						
Good *	-	-		699 (3.0%)	341 (46.5%)	
Poor, not in crisis	-	-		1136 (60.0%)	352 (48.0%)	2*10 ^**−8**^
In crisis	-	-		56 (37.0%)	40 (5.5%)	
**Postcards sent**						
No *	-	-		676 (35.7%)	301 (41.1%)	
Yes	-	-		1215 (64.3%)	432 (58.9%)	**0.007**

From data collected at the D10-D21 call, a significant relationship was found between lost to follow-up and the need of help, the patient’s state at the end of the interview, and postcards sent after the interview **Table 3.**

#### b) Multiple logistic regression

Multiple regression showed that patients at risk of being lost to follow-up in general were non-first suicide attempters (OR = 1.27), alcohol consumers (OR = 1.10), patients having non-presence of a companion on arrival at the emergency room (OR = 1.13), and patients who didn’t make or receive any calls. Those whose phone contact was unsuccessful but who still had cards sent out, are more likely to be lost to follow-up than those who had at least one successful telephone contact (OR = 1.47). On the other hand, those who have no contact and no sent cards are even more at risk of being lost to follow-up (OR = 1.58) **Table 4**.

**Table 4:** Multiple regression of LFU and non-LFU patients.

	All patients			First suicide attempters			Non-first suicide attempters		
Variables	OR	95% IC	P (OR)	OR	95% IC	P (OR)	OR	95% IC	P (OR)
**Suicide attempters** *“Non-first suicide attempters”*	1.27	1.12–1.45	**<0.001**	-	-	-	-	-	-
**Alcohol consumption** “*Yes*”	1.10	1.00–1.22	**0.049**	1.08	0.94–1.24	>0.1	1.12	0.97–1.29	>0.1
**Accompanying** *“No”*	1.13	10.0–1.26	**0.038**	1.20	1.01–1.43	0.043	-	-	-
**Duration of hospitalization stay**									
*“1 day”*	0.95	0.82–1.11	>0.1	1.0	0.87–1.32	>0.1	0.79	0.62–0.99	0.043
*“2 days”*	0.83	0.71–0.96	**0.016**	0.96	0.77–1.19	>0.1	0.67	0.53–0.85	**<0.001**
**Number of outgoing intermediate call issued successfully** *“0 call”*	1.37	1.15–1.63	**<0.001**	0.93	0.58–1.48	>0.1	1.51	1.24–1.84	**<0.001**
**Number of incoming intermediates calls** *“0 call”*	1.70	1.46–1.99	**<0.001**	2.60	1.30–5.33	0.008	1.47	1.23–1.76	**<0.001**
**No Phone contacts?**									
*“No contact but cards send”*	1.47	1.24–1.73	**<0.001**	1.69	0.78–3.58	>0.1	1.22	0.99–1.50	0.057
*“No contact No cards send”*	1.58	1.32–1.89	**<0.001**	1.83	0.84–3.88	>0.1	1.24	0.97–1.59	0.084
**Number of outgoing calls to the patient’s family and friends** *“0 call”*	1.28	1.10–1.48	**0.001**	2.11	1.63–2.72	**<0.001**	1.02	0.85–1.23	>0.1
**Number of incoming calls from the patient’s family and friends** *“0 call”*	1.36	1.07–1.73	**0.011**	1.24	0.79–1.94	>0.1	1.48	1.10–1.98	9*10^−3^
**Years**									
*“2016”*	1.36	1.19–1.55	**<0.001**	1.86	1.53–2.59	**<0.001**	1.00	0.83–1.20	>0.1
*“2017”*	4.40	3.78–5.12	**<0.001**	4.12	3.32–5.13	**<0.001**	4.51	3.65–5.60	**<0.001**
*“2018”*	2.68	2.33–3.08	**<0.001**	1.89	1.57–2.28	**<0.001**	4.27	3.45–5.29	**<0.001**
**VDI** *“Yes”*	0.91	0.78–1.05	>0.1	0.84	0.69–1.01	0.074	0.85	0.68–1.06	>0.1
**Hanging** *“Yes”*	1.08	0.84–1.40	>0.1	-	-	-	-	-	-
**Phlebotomy** *(***Self-bloodletting***) “Yes”*	-	-	-	-	-	*-*	0.74	0.56–0.99	**0.041**

Compared to 2015, the year in which VigilanS started, 2017 was the year in which the risk of being lost to follow up of was very high (OR = 4.40), followed by 2018 (OR = 2.68). Patients who spend 2 days and more in hospital were less likely to be lost to follow-up (OR = 0.83), opposed to those discharged on the same day. **Table 4.**

In non-first suicide attempters, it was found that patients who attempted suicide by phlebotomy were less likely to be lost to follow-up (OR = 0.74) **Table 4.** The variables resulting from the D10-D21 call were not significant.

## Discussion

### Main findings and comparison with findings from other studies

The interest and the originality of this study is to focus on a population that is still poorly investigated in the literature. To our knowledge, there is little research on post SA interventions implemented in real life conditions on a large population: none of them focus on the loss of follow-up.

Some of our results are consistent with the socio-demographic and clinical characteristics of suicidal patients described in previously published studies; in particular, significantly more women attempt suicide than men [33–35], and the operating mode by VDI [33, 36–40]. The proportion of patients with a history of one or more SA at inclusion in VigilanS was approximately 47%. This indicates that our sample had a relatively high proportion of patients with an increased risk of suicide at the first stay.

We have identified that non-primary-suicide attempters are more likely to be lost to follow-up. According to the literature, patients with a history of previous suicide attempts have a higher risk of fatal suicidal act during their next attempt [3]. This may explain the loss of follow-up in this group of patients. Other studies have pointed out that people with a history of suicide are more likely to have ineffective coping strategies such as avoidance, emotional adjustment, self-accusation, and a preference to solve problems on their own [41, 42]. It is possible that these behaviors may explain the loss of follow-up of multi-suicidal patients during follow-up.

According to several studies, alcohol use is an important factor in suicidal risk. Almost a quarter of suicide deaths are directly attributable to alcohol [43], which is often used during suicide attempts (both non-lethal and fatal) [44–46]. We also found that this factor (alcohol consumption) should also be taken into account in patient follow-up, as our multivariate analysis shows compared to those who do not consume alcohol. We also identified that patients who didn’t have a companion on arrival at the emergency room are more likely to be lost than patients having a companion on arrival at the emergency room. According to Luoma et al, the suicide rate for single persons is twice as high as that for married persons and four to five times higher for separated, divorced and widowed persons [47]. Having someone close to you can also be considered an important factor in reducing the LFU.

In terms of length of hospitalization, patients spent an average of one day in hospital. However, the longer the hospital stay, the lower the risk of losing follow-up. Patients who spent 2 or more days in hospital are about 15% less likely to be lost to follow-up in all subjects and about 50% less likely to be loss to follow-up in non-first suicide attempters, in contrast to those who left the hospital on the same day. According to Pushpakumara et al, a longer hospital stay could have an effect on reducing suicidal recurrence by providing a safer environment during this high-risk post-attempted period [48], and thus lead the patient to continue the previous day’s suicide project until completion.

Regarding phone calls, incoming intermediate calls are usually long calls from patients in need of help and/or in need of a listening ear, and outgoing calls are often for certain interventions, or for patients who could not be reached on previous calls. Regardless of the type of incoming or outgoing call, there is a risk of suicide among these patients, which may explain the risk of loss to follow-up during the monitoring.

Concerning the 6^th^ month call, our study showed that the call planned at 6^th^ month marking the end of the follow-up in VigilanS, often did not occur at 6th month as planned. They were carried out later, with an average time of about 8 months.

The year 2015 is the year when there is less risk of loss of follow-up, unlike other years. The year 2015 is the starting year of VigilanS, and it can be seen that as the years progress, there is a great increase in the burden on VigilanS staff, which has surely made contact management and follow-up difficult, and eventually subsequent loss of follow-up. However, the positive point in this study is that there is a decrease in the risk of losing follow-up after a peak in 2017. This shows improvement of the system over time.

The method of attempted suicide by phlebotomy has been identified as a protective factor in multi-suicidal patients. Phlebotomy, also known as a self-mutilation, is defined as the deliberate and direct destruction or alteration of a vein without conscious suicidal intent, by draining oneself of one’s own blood through a venipuncture or intravenous cannula [31, 32]. Some research suggests that self-harm is a common reaction to social isolation and fear [49, 50]. According to Favazza et al, episodes of self-harm are often followed by feelings of disappointment or abandonment [51], feelings of anger, upset, or loneliness [52]. The most frequently cited reason for antecedent self-harm is psychological distress, between 40% [53] and 64% of cases [54]. This shows the importance of maintaining contact after a suicide attempt, particularly in multi-suicidal patients, through the telephone call at D10-D21, and may explain this protective role against loss of follow-up in these self-harming patients.

### Strengths and weaknesses

Loss of follow-up is one of the difficulties encountered in many research studies. Faced with this difficulty, it is important to know what characterizes patients lost to follow-up. Our study is the only one that compares the characteristics of suicidal patients lost to follow-up with that of patients not lost to follow-up patients, all of whom are monitored by a post attempt monitoring system. One problem of studies with follow-up is missing data. Most patients did not complete their 6-month follow-up, and therefore data not collected during follow-up may bias the results of our analyses, such as the estimate of average time call at 6 months.

In addition, our study was based only on the hospital environment, and a part of SA in the population does not lead to hospitalization. In France, however the proportion of non-hospitalized SA is fairly low, around 8%, but our results cannot be generalized to the whole population [55]. Nevertheless, the quality of our sample is extremely close to the reality of the clinical field, since it is an exhaustive sample of patients in real life conditions.

Our work has important implications for preventive interventions, in order to improve the follow-up strategy for patients leaving hospital after a SA.

To conclude, the presence of substantial loss to follow-up calls for special attention towards non-first suicide attempters, alcohol consumers, patients who didn’t have a companion on arrival at the emergency room, patients with unsuccessful or who didn’t make the calls. One of an important finding is the length of hospitalization, which has an important clinical implication in the LFU, by prolonging the hospital stay with one day or more. The results of our study provide us with a valuable insight into the profiles of patients likely to be incompletely monitored, which is important for suicide prevention in general.

## Supporting information

S1 Table List of variables. (DOCX)

S1 Fig Patient pathway of loss of follow-up during the first 4 stays in VigilanS. ^a^ NC: Not concerned by stay. ^**b**^ Of the 8335 patients lost to follow-up during the first stay, 308 patients had a suicide reattempt and were followed up a second time in VigilanS, of which 284 patients were lost to follow-up and 24 were followed up until the end of the monitoring.
(TIF)

S1 Data (CSV)

S2 Data General data. (CSV)

## Abbreviations

BCI: Brief Contact Interventions
D10-D21: Call between the 10^th^ and 21^st^ days after SA
LFU: lost to follow-up
OR: Odds Ratio
P: P-value
SA: Suicide Attempt
SD: Standard Deviation
SUA: statistical units of analysis
VDI: Voluntary Drug Intoxication
WHO: World Health Organization
