# Supplementary material for: Loss to follow-up in a population-wide brief contact intervention to prevent suicide attempts - The VigilanS program, France
Source: PLoS One. 2022 Mar 1;17(3):e0263379. doi: 10.1371/journal.pone.0263379 (PMC8887722; doi:10.1371/journal.pone.0263379)
Supplement: S1 Table — (DOCX) [file pone.0263379.s001.docx]

**S1 Table: List of variables**

**General variables**

| **Age** | Age at entry into ViglanS |
| --- | --- |
| **Sex** | Gender of patient (Male / Female) |
| **Geographic sub region (French “Departement”)** | Department in which the patient lives (North; Pas-de-Calais; others). |
| **Date SA** | Date of the patient's SA when entering ViglanS |
| **Suicide attempters** | If the patient is on his first suicide attempt (First suicide attempters) or a reiteration (Non-first suicide attempters). |
| **Alcohol consumption** | If the patient consumes alcohol regularly on a daily basis (Yes/No) |
| **Accompanying person** | Whether the patient has a companion on arrival at the emergency room (Yes/No) |
| **Duration of hospitalization stay** | Length of time spent in hospital following a SA (0 day, 1 day, 2 days and more). |
| **Outgoing D10-D21 call issued successfully?** | If calls made at D10-D21 were successful (Yes/No), for non-first suicide attempters. |
| **Outgoing 6M call issued successfully?** | Whether the 6-months call went through (Yes/No) |
| **Outgoing intermediate call issued successfully?** | If an intermediate call was made during the follow-up (Yes/No) |
| **Number of intermediate outgoing calls issued successfully** | Number of outgoing intermediate calls made to the patient and successfully completed (0 call received; 1 call received and more) |
| **Number of incoming calls from the patient** | Number of intermediate calls received during the follow-up (0 call received; 1 call received and more) |
| **Phone contact** | Whether a patient has had outgoing calls issued successfully or incoming calls (In contact) or no contact but has had postcards sent (No contact but cards send), or neither (No contacts No cards send). |
| **Number of outgoing call to the patient's family and friends** | Number of intermediate calls made to the entourage: This is the number of calls made to the entourage during the follow-up (0 call received; 1 call received and more) |
| **Number of incoming call from the patient's family and friends** | Number of calls received from the entourage: This is the number of calls received from the entourage during the follow-up (0 call received; 1 call received and more) |
| **Means of SA** | This is the type of means used by the patient to carry out his SA (Voluntary Drug Intoxication or VDI; Hanging; Phlebotomy; others (Firearm, Injury, Drowning, and Jump). |

**Variables at D10-D21 phone call (for non-first suicide attempters)**

| **Evolution of the discomfort** | Evolution of the discomfort from the time of entry into ViglanS until the time of the call D10-D21 (Stationary; Favorable; Unfavorable). |
| --- | --- |
| **Patient’s state at the end of the interview** | Patient's state at the end of the interview D10-D21 (Good; Poor, not in crisis; In crisis). |
| **Need help** | Whether the patient needed help during this call D10-D21 (Yes/No) |
| **Follow-up by a Psychiatrist** | If the patient is being followed by a Psychiatrist during their VigilanS enrollment (Yes/No). |
| **Postcards sent** | Whether the patient required postcards to be sent after this telephone call D10-D21 (Yes/No). |
