# Supplementary figures and images for: Loss to follow-up in a population-wide brief contact intervention to prevent suicide attempts - The VigilanS program, France
Source: PLoS One. 2022 Mar 1;17(3):e0263379. doi: 10.1371/journal.pone.0263379 (PMC8887722; doi:10.1371/journal.pone.0263379)

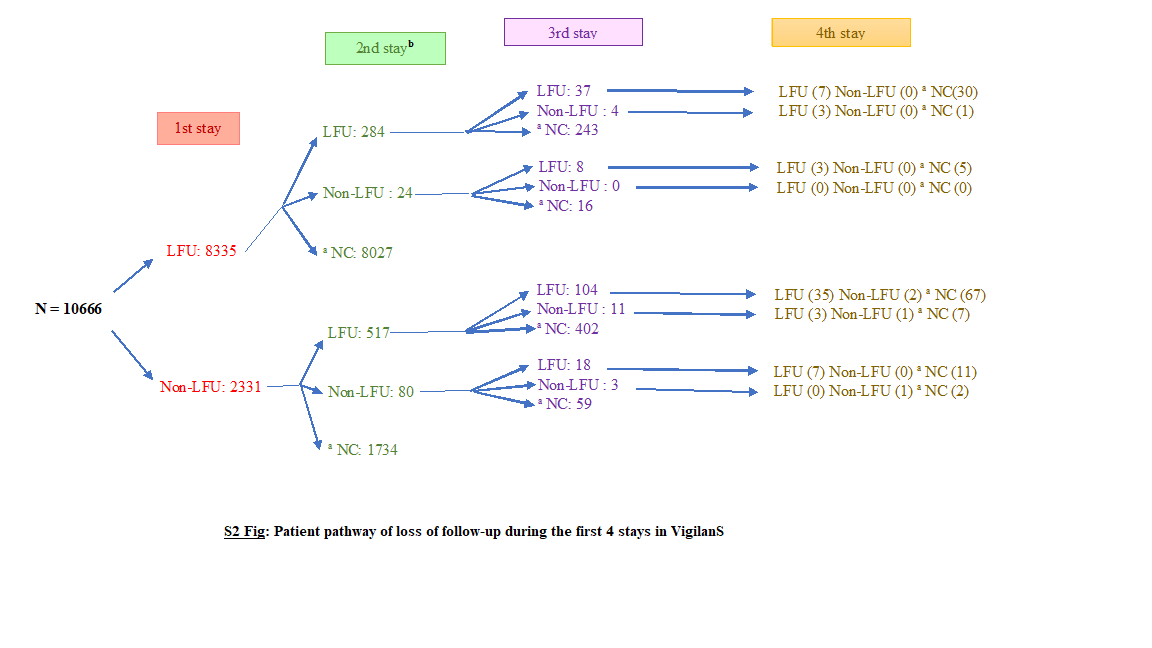

Supplement: S1 Fig — a NC: Not concerned by stay. b Of the 8335 patients lost to follow-up during the first stay, 308 patients had a suicide reattempt and were followed up a second time in VigilanS, of which 284 patients were lost to follow-up and 24 were followed up until the end of the monitoring. (TIF) [file pone.0263379.s002.tif]
